# Supplementary material for: Regrowth-delay body as a bacterial subcellular structure marking multidrug-tolerant persisters
Source: Cell Discov. 2019 Jan 22;5:8. doi: 10.1038/s41421-019-0080-3 (PMC6341109; doi:10.1038/s41421-019-0080-3)
Supplement: Supplementary file 1 — Supplementary Information [file 41421_2019_80_MOESM1_ESM.pdf]

Supplementary information

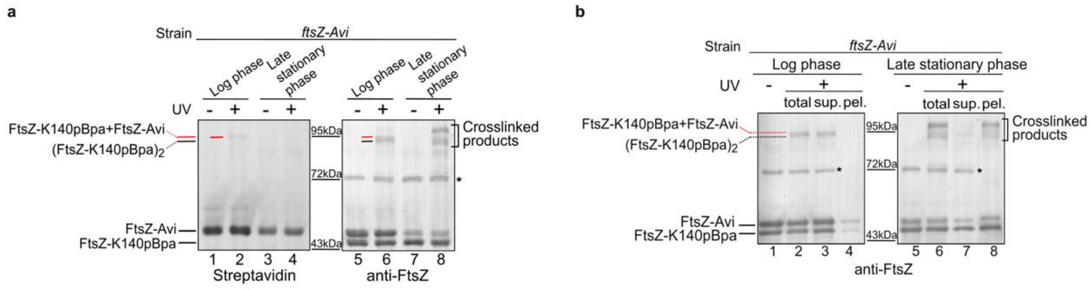

Figure S1. Unnatural amino acid mediated photo-crosslinking analysis of the FtsZ-K140pBpa variant in the log-phase and late stationary-phase cells.

(a) Blotting results for the detection of photo-crosslinked products of the FtsZ-K140pBpa variant in the log-phase and late stationary-phase *ftsZ-Avi* cells exposed to UV light, as probed with streptavidin-alkaline phosphate conjugate (left part) or antibodies against FtsZ (right part). The asterisk indicates a non-specific protein band detected when probed with the anti-FtsZ antibodies. **(b)** Immunoblotting results for the detection of photo-crosslinked products of the FtsZ-K140pBpa variant, as well as the free FtsZ monomers, in the supernatant (sup.) and pellet (pel.) fractions of the log-phase or late stationary-phase *ftsZ-Avi* cells, as probed with antibodies against FtsZ. Positions of the FtsZ monomers and photo-crosslinked dimers are shown on the left of the gels, positions of the molecular weight markers are shown in the middle of the gels.

Here, to verify the reported *in vitro* assembly pattern of FtsZ protofilaments in *E. coli* cells, we performed *in vivo* protein photo-crosslinking analysis by replacing the amino acid residue K140, located at the longitudinal interface of the FtsZ protofilament, with the unnatural amino acid pBpa. This FtsZ-K140pBpa variant, which we demonstrated

to be able to support cell division in the absence of wild-type FtsZ, was then heterologously expressed in a strain whose genomic *ftsZ* gene was modified to encode an Avi-tagged FtsZ variant (the Avi tag could be specifically probed with streptavidin). As shown by the blotting results displayed here, the FtsZ dimers were formed either between the FtsZ-K140pBpa and FtsZ-Avi monomers (thus detectable not only by streptavidin AP conjugate but also by antibodies against FtsZ; red arrows) or between two FtsZ-K140pBpa monomers (only detectable by antibodies against FtsZ; black arrows) in actively dividing log-phase cells (lanes 2 and 6). These observations confirmed the location of residue K140 at a self-assembling interface and that FtsZ assembles into homo-oligomers in log-phase cells. By contrast, in late stationary-phase cells, the photo-crosslinked FtsZ dimers became no longer detectable, instead, multiple photo-crosslinked products between FtsZ-K140pBpa and other proteins were readily detected (lane 8).

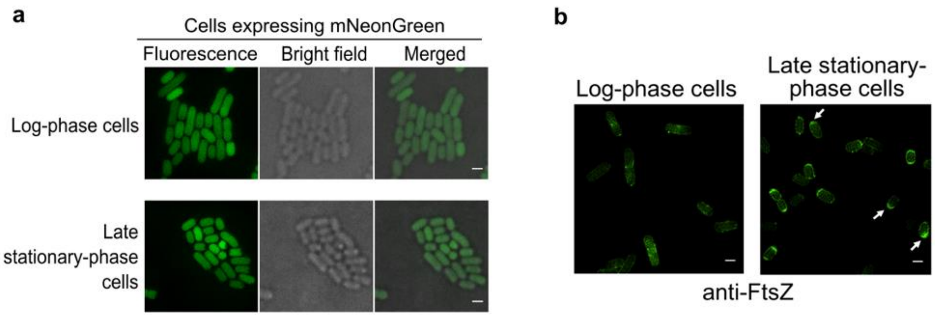

Figure S2. Fluorescence microscopic images of the log-phase and late stationary-phase cells in which mNeonGreen (without being fused to FtsZ) was heterologously expressed (a); the immunofluorescent analysis against FtsZ for the log-phase and late stationary-phase cells (b). Scale bars, 1 μm.

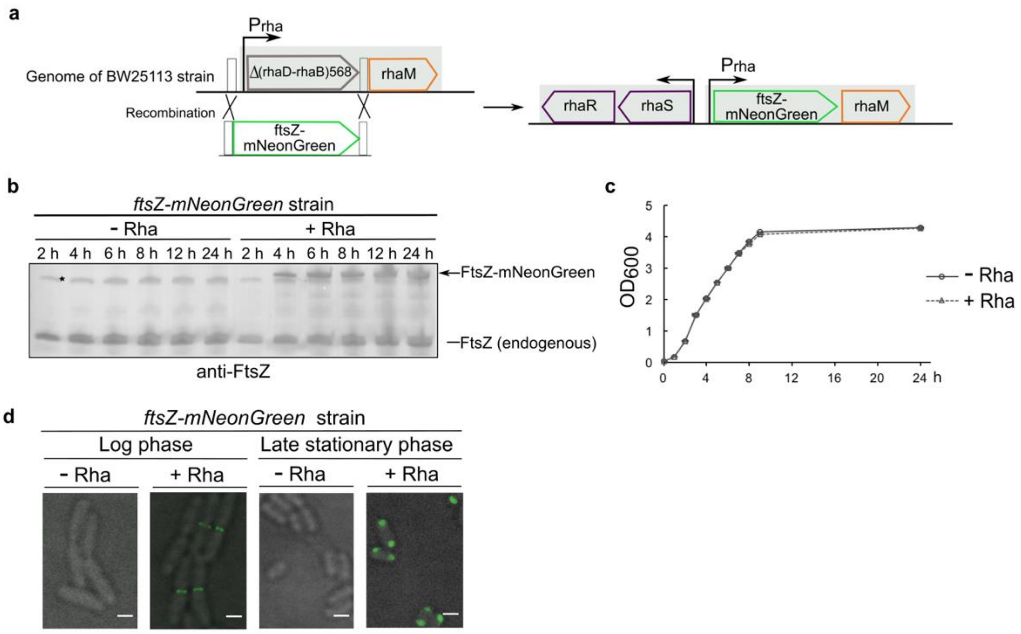

Figure S3. Construction and verification of the *ftsZ-mNeonGreen* strain.

(a) The *ftsZ-mNeonGreen* strain was constructed by replacing part of the rhamnose operon by the *ftsZ-mNeonGreen* gene (green outline) in the *E. coli* genome. The transcription initiation sites and directions of transcriptions are both indicated by the arrows (top panel). **(b)** Immunoblotting results for detecting the FtsZ-mNeonGreen protein expressed in the *ftsZ-mNeonGreen* strain as cultured in the presence (+Rha) or absence (-Rha) of rhamnose (0.02%) to the indicated time points, as probed with antibodies against FtsZ; positions of the two forms of FtsZ are indicated on the right. Asterisk indicates a non-specific protein band (bottom left panel). **(c)** Growth curves of the *ftsZ-mNeonGreen* strain cultured in the presence (+Rha) or absence (-Rha) of rhamnose (0.02%), as prepared by measuring the OD_{600} values at the indicated time points (bottom right panel). **(d)** Bright field and fluorescence microscopic images of the log-phase or late stationary-phase *ftsZ-mNeonGreen* cells cultured in LB medium with (+Rha) or without (-Rha) the addition of rhamnose. Scale bars, 1 μm.

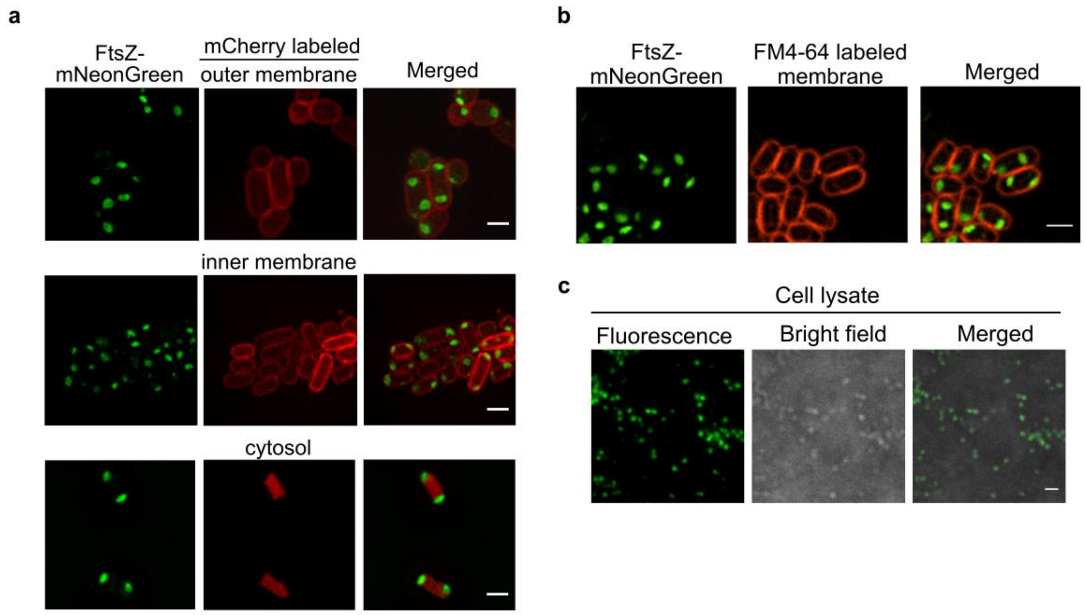

Figure S4. Cellular localization of the FtsZ granules.

(a) Fluorescence microscopic images of the late stationary-phase *ftsZ-mNeonGreen* cells whose outer membrane (top), inner membrane (middle) or cytosol (bottom) was separately labeled with OmpA-fused mCherry, NlpA anchoring peptide-fused mCherry or unfused mCherry, respectively. Scale bars, 1 μm. **(b)** Fluorescence microscopic images of the late stationary-phase *ftsZ-mNeonGreen* cells stained with the membrane specific FM4-64 dye. Scale bars, 1 μm. **(c)** Fluorescence and bright field microscopic images of the FtsZ granules detected in the lysates of late stationary-phase (cultured to 24 h) *ftsZ-mNeonGreen* cells. Scale bars, 1 μm.

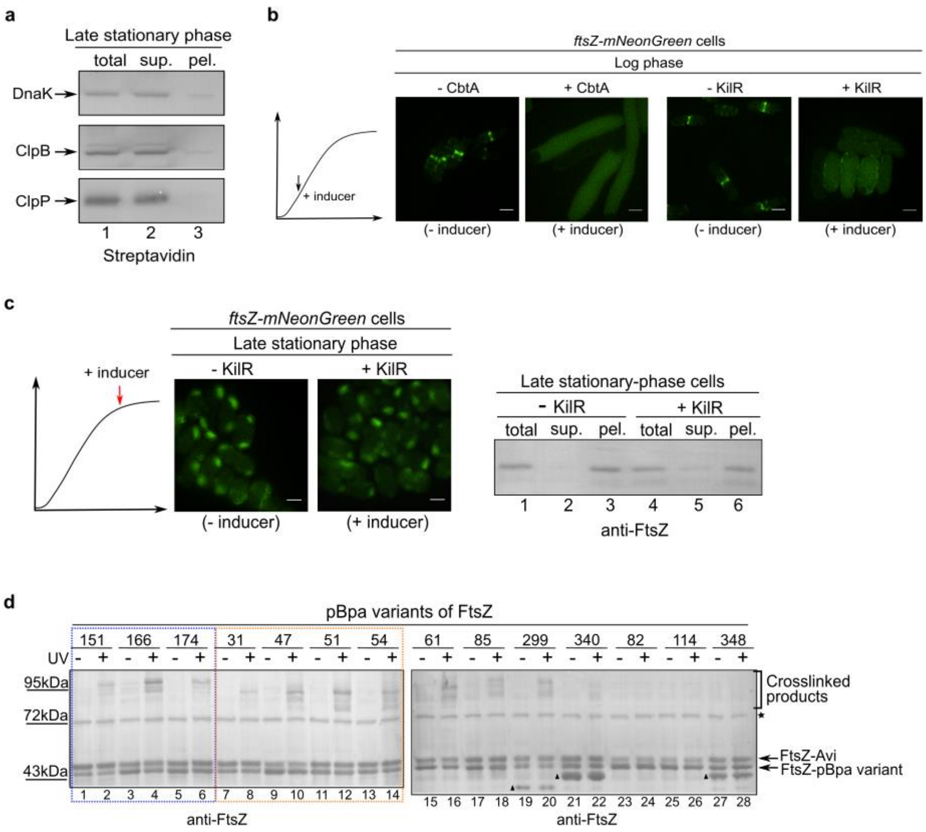

Figure S5. Analysis for the folding status of FtsZ in the cell-pole granules.

(a) Blotting results for the detection of DnaK-Avi, ClpB-Avi or ClpP-Avi protein in the indicated fractions of late stationary-phase *E. coli* cells, probed with the streptavidin-AP conjugate (against the Avi tag). **(b)** Fluorescence microscopic images of log-phase *ftsZ-mNeonGreen* cells in which the expression of the CbtA or KilR inhibitor protein was induced. Scale bars, 1 μm. **(c)** Fluorescence microscopic images of the late stationary-phase *ftsZ-mNeonGreen* cells in which the expression of KilR was induced (left panel) and the corresponding immunoblotting results for the detection of the indicated cell lysate fractions, probed with antibodies against FtsZ (right panel). Scale bars, 1 μm. **(d)** Immunoblotting results for the detection of photo-crosslinked products of the indicated pBpa variants of FtsZ in late stationary-phase *ftsZ-Avi* cells probed with antibodies against FtsZ. The asterisk indicates a non-specific band, and the

triangles indicate truncated forms of FtsZ that were produced due to failed pBpa incorporation.

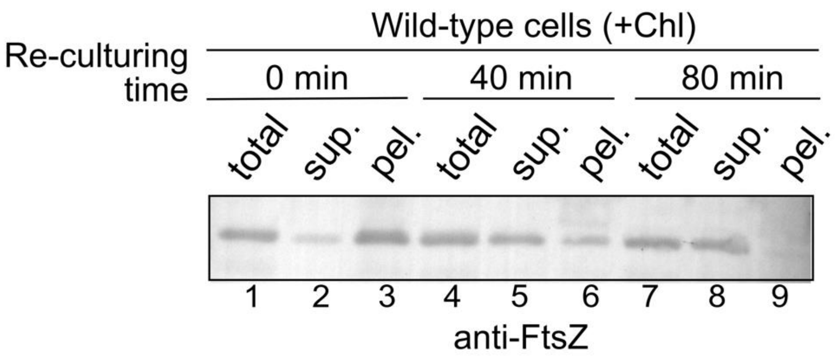

Figure S6. Cellular distribution of the FtsZ proteins for the late stationary-phase cells being re-cultured in the presence of chloramphenicol.

Immunoblotting results for the detection of FtsZ in the indicated cell lysate fractions when late stationary-phase wild-type cells were re-cultured in fresh LB medium containing chloramphenicol to the indicated time points, probed with anti-FtsZ antibodies.

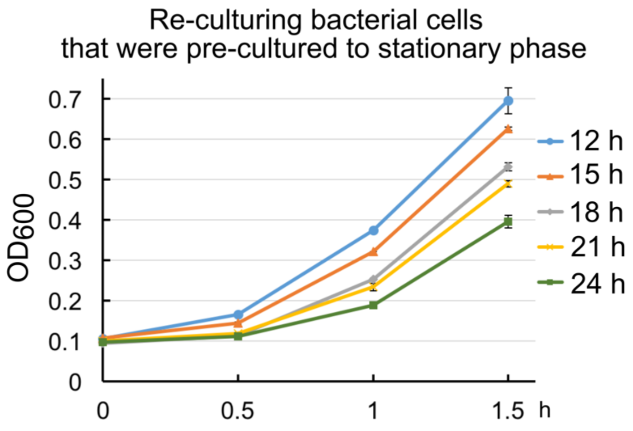

Figure S7. Growth curves of the re-cultured wild-type *E. coli* cells that were pre-cultured to the indicated time points in the stationary phase.

These growth curves were used to calculate the average initial doubling time upon re-division (re-division T_{id}), which reflects the regrowth lag time for each set of the non-growing stationary-phase cells. At least three biological replicates were analyzed for obtaining each value.

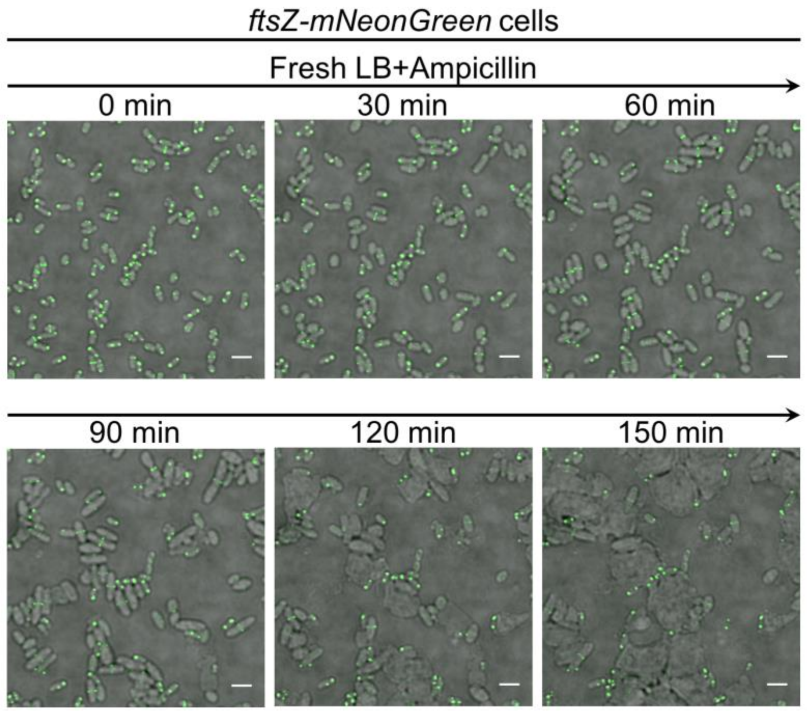

Figure S8. Live-cell imaging analysis for the late stationary-phase *ftsZ-mNeonGreen* cells being re-cultured in fresh medium in the presence of ampicillin to the indicated time points. Scale bars, 3 μm.

a

	as photo-crosslinked products of FtsZ	as regrowth-delay body pellet
**Cell division proteins**	FtsA, MinD, MreB	FtsZ, FtsA, MinD, MreB, ZapC, FtsE, FtsX, MinE
**Metabolic enzymes**	PGK (Phosphoglycerate kinase), GadB (Glutamate decarboxylase B), NuoC (Subunit of complex I in respiratory chain), SdhA (Subunit of Complex II in respiratory chain), AtpD (β subunit of ATP synthase), PtsI (PTS enzyme I)	PGK (Phosophoglycerate kinase), GadB (Glutamate decarboxylase B), NuoF (Subunit of complex I in respiratory chain), SdhB (Subunit of complex II in respiratory chain), AceE (Pyruvate dehydrogenase E1 subunit), SucC (β subunit of succinyl-CoA synthetase), Mdh (Malate dehydrogenase), AceA (Isocitrate lyase)
**Subunits of RNA polymerase**	RpoS (sigmaS factor)	RpoS (sigmaS factor), RpoA (α subunit), RpoB (β subunit), RpoC (β' subunit)
**Aminoacyl tRNA synthetases**		TrpS (Tryptophan-tRNA ligase), LysS (Lysine-tRNA ligase), CysS (Cysteine-tRNA ligase), AsnS (Asparagine-tRNA ligase), ProS (Proline-tRNA ligase), SelA (Selenocysteine synthase)
**Ribosomal proteins**		RpsE (30S ribosomal subunit S5), RbfA (30S ribosome binding factor), RplA (50S ribosomal subunit L1), RplJ (50S ribosomal subunit L10)

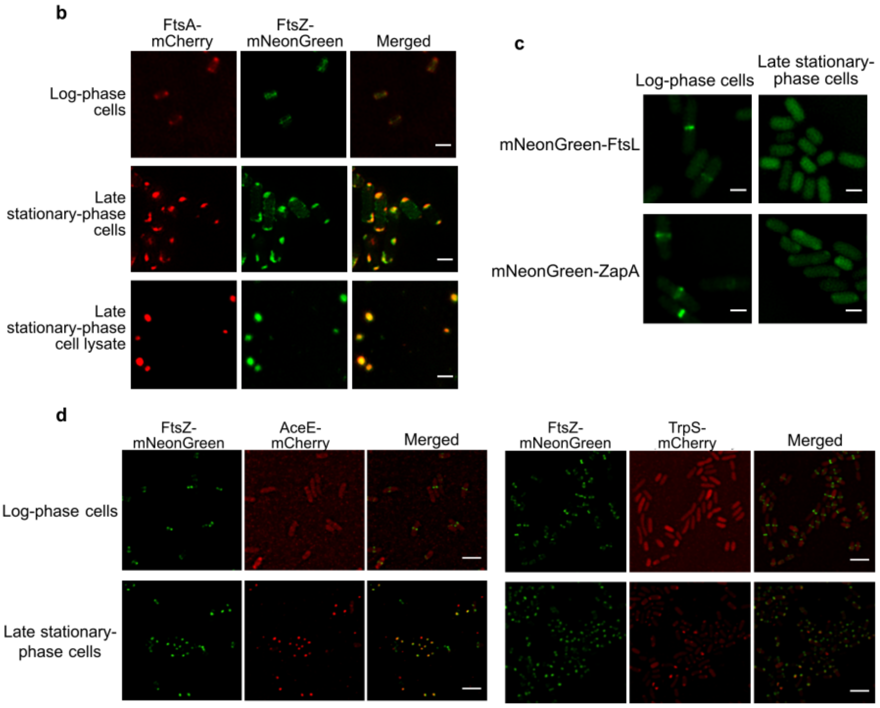

Figure S9. Identification for the components in the regrowth-delay bodies and detection for the localization of several identified ones.

(a) List of major proteins identified by mass spectrometry analyses, both as the photo-crosslinked products of five pBpa variants of FtsZ and as present in the pellets containing the regrowth-delay bodies, isolated from the *ftsZ-Avi* and wild-type late stationary-phase cells, respectively. **(b)** Fluorescence microscopic images of the log-phase or late stationary-phase *ftsZ-mNeonGreen* cells in which FtsA-mCherry was

expressed from a plasmid controlled by a constitutive promoter, as well as of the lysate of these late stationary-phase cells. Scale bars, 1 μm. **(c)** Fluorescence microscopic images of the log-phase or late stationary-phase cells in which mNeonGreen-FtsL or mNeonGreen-ZapA was heterogeneously expressed from a plasmid controlled by a constitutive promoter. Scale bars, 1 μm. **(d)** Fluorescence microscopic images of the log-phase or late stationary-phase cells in which AceE-mCherry or TrpS-mCherry was heterogeneously expressed from a plasmid. Scale bars, 3 μm.

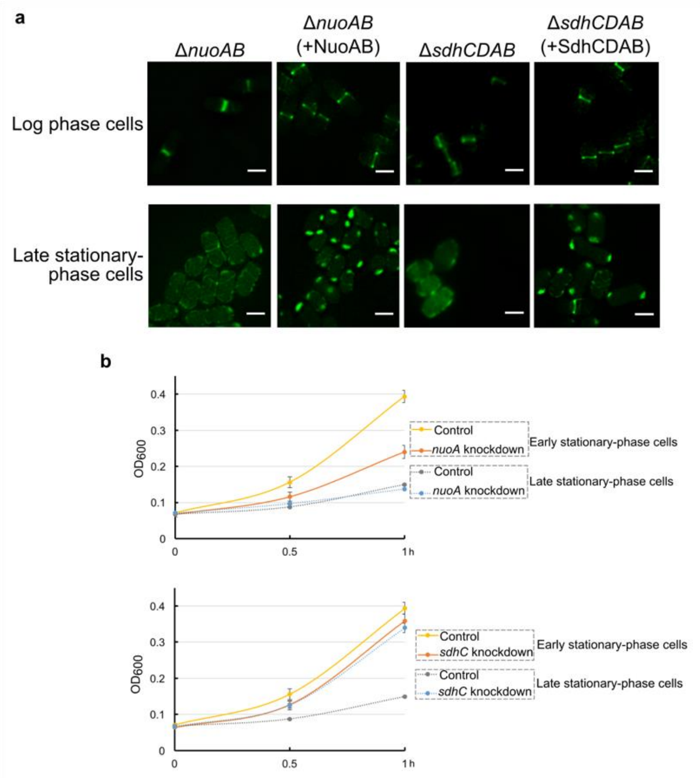

Figure S10. Fluorescence imaging analysis of the *nuoAB* knockout (Δ*nuoAB*) or *sdhCDAB* knockout (Δ*sdhCDAB*) cells, and the growth curves of the *nuoA* or *sdhC* knockdown cells.

(a) Fluorescence microscopic images of log-phase (top) and late stationary-phase (bottom) *ftsZ-mNeonGreen* cells in which the *nuoAB* or *sdhCDAB* genes were deleted. Scale bars, 1 μm. **(b)** Growth curves of the re-cultured early or late stationary-phase *nuoA* or *sdhC* knockdown cells. Here, cells in which a non-targeting crRNA was expressed from a plasmid were analyzed as the control. All experiments were independently repeated three times.

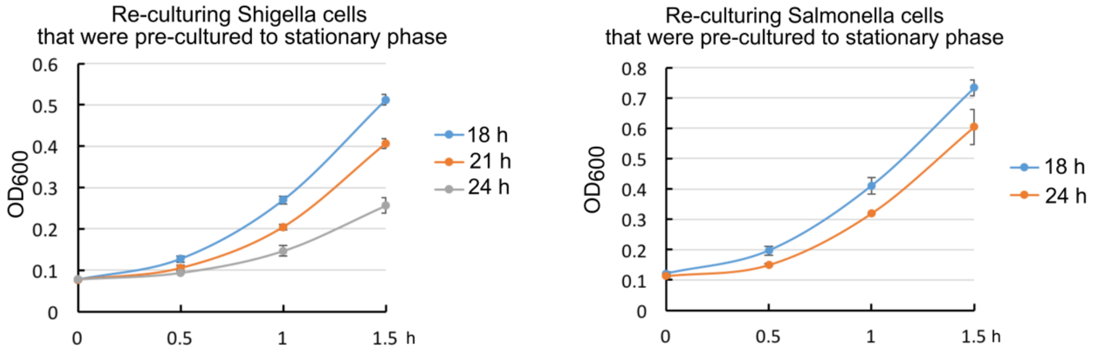

Figure S11. Growth curves of the re-cultured *Shigella* and *Salmonella* bacterial cells that were pre-cultured to the indicated time points in stationary phase.

Table S1. *E. coli* strains used in this study

Strain	Genotype[a]	Source/Reference
BW25113	Δ(*araD-araB*)567 Δ*lacZ*4787(::*rrnB*-3) *rph*-1 Δ(*rhaD-rhaB*)568 *hsdR*514	[1]
LY928	BW25113 Δ*insH11*::aminoacyl-tRNA synthetase of pBpa-$tRNA^{pBpa}$	Laboratory storage[2]
ftsZ-Avi	LY928 *ftsZ*::*ftsZ-Avi tag*	Laboratory storage
ftsZ-mNeonGreen	LY928 Δ(*rhaD-rhaB*)568::*ftsZ-mNeonGreen*	Recombineering
ftsZ-mNeonGreen-dnaK-mCherry	*ftsZ-mNeonGreen dnaK*::*dnaK-mCherry*	Recombineering
ftsZ-mNeonGreen-clpB-mCherry	*ftsZ-mNeonGreen clpB*::*clpB-mCherry*	Recombineering
Δ*cas3*	LY928 Δ*cas3* P_{casA}::P_{con}	Recombineering[3]
*ftsZ-mNeonGreen-*Δ*cas3*	Δ*cas3* Δ(*rhaD-rhaB*)568::*ftsZ-mNeonGreen*	Recombineering
Δ*nuoAB*	*ftsZ-mNeonGreen* Δ*nuoAB*::Kan^R	Recombineering
Δ*sdhCDAB*	*ftsZ-mNeonGreen* Δ*sdhCDAB*::Kan^R	Recombineering
Salmonella	*Salmonella* Typhimurium SL1344	ATCC
Shigella	*Shigella flexneri* serotype 2a 2457T	ATCC

[a] P_{con} is a synthetic constitutive promoter[3].

Table S2. Plasmids used in this study

Plasmid	Genotype[a]	ori	Reference/Source
pTet-FtsZ-pBpa-mNeonGreen	*bla* P_{tet1}::*ftsZ-pBpa-mNeonGreen*	pBR322	This study
pTet-mNeonGreen	*bla* P_{tet1}::*mNeonGreen*	pBR322	This study
pTac-mCherry	*bla* P_{con}::*mCherry*	pBR322	This study
pBAD-SSnlpA-mCherry	*bla* P_{ara}::*signal peptide of nlpA-mCherry*	pBR322	This study
pBAD-OmpA-mCherry	*bla* P_{ara}::*ompA-mCherry*	pBR322	This study
pACE	*cl* P_{ara}::λ-Red recombinase P_{ara}::I-SceI endonuclease	p15A	Laboratory storage[4]
pYLC-rha-FtsZ-mNeonGreen	*bla* upstream homologous sequence-*ftsZ-mNeonGreen-Kan*^{R}-downstream homologous sequence, for inserting *ftsZ-mNeonGreen* into the genomic rha operon	pBR322	This study
pYLC-dnaK-mcherry	*bla* upstream homologous sequence-*mCherry-Kan*^{R}-downstream homologous sequence, for inserting *mCherry* into the C-terminus of genomic *dnaK*	pBR322	This study
pYLC-clpB-mcherry	*bla* upstream homologous sequence-*mCherry-Kan*^{R}-downstream homologous sequence, for inserting *mCherry* into the C-terminus of genomic *clpB*	pBR322	This study
pltetO-CbtA	*bla* $P_{tet\text{-}T}$::*cbtA-coulping-mCherry-his*	pBR322	This study
pTet-FtsZ-140pBpa	*bla* P_{tet1}::*ftsZ-140pBpa*	pBR322	Laboratory storage
pTet-FtsZ-31pBpa	*bla* P_{tet1}::*ftsZ-31pBpa*	pBR322	Laboratory storage
pTet-FtsZ-47pBpa	*bla* P_{tet1}::*ftsZ-47pBpa*	pBR322	Laboratory storage

pTet-FtsZ-51pBpa	*bla* P$_{tet1}$::*ftsZ-51pBpa*	pBR322	Laboratory storage
pTet-FtsZ-54pBpa	*bla* P$_{tet1}$::*ftsZ-54pBpa*	pBR322	Laboratory storage
pTet-FtsZ-61pBpa	*bla* P$_{tet1}$::*ftsZ-61pBpa*	pBR322	Laboratory storage
pTet-FtsZ-82pBpa	*bla* P$_{tet1}$::*ftsZ-82pBpa*	pBR322	Laboratory storage
pTet-FtsZ-85pBpa	*bla* P$_{tet1}$::*ftsZ-85pBpa*	pBR322	Laboratory storage
pTet-FtsZ-114pBpa	*bla* P$_{tet1}$::*ftsZ-114pBpa*	pBR322	Laboratory storage
pTet-FtsZ-151pBpa	*bla* P$_{tet1}$::*ftsZ-151pBpa*	pBR322	Laboratory storage
pTet-FtsZ-166pBpa	*bla* P$_{tet1}$::*ftsZ-166pBpa*	pBR322	Laboratory storage
pTet-FtsZ-174pBpa	*bla* P$_{tet1}$::*ftsZ-174pBpa*	pBR322	Laboratory storage
pTet-FtsZ-288pBpa	*bla* P$_{tet1}$::*ftsZ-288pBpa*	pBR322	Laboratory storage
pTet-FtsZ-340pBpa	*bla* P$_{tet1}$::*ftsZ-340pBpa*	pBR322	Laboratory storage
pTet-FtsZ-348pBpa	*bla* P$_{tet1}$::*ftsZ-348pBpa*	pBR322	Laboratory storage
pTac-trpS-Avi	*bla* P$_{con}$::*trpS-Avitag*	pBR322	This study
pTac-rpoS-Avi	*bla* P$_{con}$::*rpoS-Avitag*	pBR322	This study
pTac-aceE-Avi	*bla* P$_{con}$::*aceE-Avitag*	pBR322	This study
pTac-rplL-Avi	*bla* P$_{con}$::*rplL-Avitag*	pBR322	This study
pTac-FtsA-mNeonGreen	*bla* P$_{con}$::*ftsA-mNeonGreen*[5]	pBR322	This study
pTac-FtsA-mCherry	*bla* P$_{con}$::*ftsA-mCherry*[6]	pBR322	This study
pTac-mNeonGreen-ZapA	*bla* P$_{con}$::*zapA-mNeonGreen*	pBR322	This study
pTac-ZapC-mNeonGreen	*bla* P$_{con}$::*zapC-mNeonGreen*	pBR322	This study
pTac-AceE-mCherry	*bla* P$_{con}$::*aceE-mCherry*	pBR322	This study
pTac-TrpS-mCherry		pBR322	This study

	bla P_{con}::*trpS-mCherry*		
pTac-mNeonGreen-FtsL	*bla* P_{con}:: *mNeonGreen-ftsL*[7]	pBR322	This study
pYLC-Δ*Cas3*-kana	*bla* upstream homologous sequence-*Kan*^{R}-P_{con}-downstream homologous sequence, for deleting the *cas3* gene and replacing the native promoter of the Cascade operon with a constitutive promoter	pBR322	This study
pTac-nuoAi	*cat* P_{con}::crRNA sequence targeting *nuoA*	pBR322	This study
pTac-sdhCi	*cat* P_{con}::crRNA sequence targeting *sdhC*	pBR322	This study
pYLC-Δ*nuoAB*-kana	*bla* upstream homologous sequence-*Kan*^{R}-downstream homologous sequence, for deleting the *nuoAB* gene	pBR322	This study
pYLC-Δ*sdhCDAB*-kana	*bla* upstream homologous sequence-*Kan*^{R}-downstream homologous sequence, for deleting the *sdhCDAB* gene	pBR322	This study
pTac-NuoAB	*bla* P_{con}::*nuoAB*	pBR322	This study
pTac-sdhCDAB	*bla* P_{con}::*sdhCDAB*	pBR322	This study

[a] P_{tet}, P_{ara} and P_{con} indicate the Tet-on/Tet-off, arabinose and synthetic constitutive (selected from the Anderson promoter collection: parts.igem.org/Promoters/Catalog/Anderson) promoters, respectively. P_{tet1} indicates that the expression of proteins was not induced by anhydrotetracycline, just via leaky expression. $P_{tet\text{-}T}$ indicates that the λt1 transcriptional terminator was inserted before the Tet promoter to achieve a stringent expression.

Reference

1. Baba, T. *et al.* Construction of Escherichia coli K-12 in-frame, single-gene knockout mutants: the Keio collection. *Mol. Syst. Biol.* **2**, 2006.0008 (2006).

2. Wang, Y. *et al.* A Supercomplex Spanning the Inner and Outer Membranes Mediates the Biogenesis of β-Barrel Outer Membrane Proteins in Bacteria. *J. Biol. Chem.* **291**, 16720–16729 (2016).
3. Luo, M. L., Mullis, A. S., Leenay, R. T. & Beisel, C. L. Repurposing endogenous type I CRISPR-Cas systems for programmable gene repression. *Nucleic Acids Res.* **43**, 674–681 (2015).
4. Lee, D. J. *et al.* Gene doctoring: a method for recombineering in laboratory and pathogenic Escherichia coli strains. *BMC Microbiol.* **9**, 252 (2009).
5. Durand-Heredia, J. M., Yu, H. H., De Carlo, S., Lesser, C. F. & Janakiraman, A. Identification and Characterization of ZapC, a Stabilizer of the FtsZ Ring in Escherichia coli. *J. Bacteriol.* **193**, 1405–1413 (2011).
6. Ma, X., Ehrhardt, D. W. & Margolin, W. Colocalization of cell division proteins FtsZ and FtsA to cytoskeletal structures in living Escherichia coli cells by using green fluorescent protein. *Proc. Natl. Acad. Sci.* **93**, 12998–13003 (1996).
7. Ghigo, J. M. & Beckwith, J. Cell division in Escherichia coli: role of FtsL domains in septal localization, function, and oligomerization. *J. Bacteriol.* **182**, 116–129 (2000).
